# Supplementary material for: One of these things is not like the other: Mixed predator cues result in lopsided phenotypic responses in a Neotropical tadpole
Source: PLoS One. 2023 May 23;18(5):e0285968. doi: 10.1371/journal.pone.0285968 (PMC10204971; doi:10.1371/journal.pone.0285968)

VASSAR COLLEGE
DEPARTMENT OF BIOLOGY
124 Raymond Ave.
Poughkeepsie, New York 12604-0731

March 6, 2023

Dr. Justin C. Touchon
Assistant Professor
Vassar College

Dr. Peter Eklöv
Professor at Department of Ecology and Genetics
Uppsala University

Dear Dr. Eklöv,

Thank you for the recent reviews of our manuscript "One of these things is not like the other: mixed predator cues result in lopsided phenotypic responses in a Neotropical tadpole" which was submitted for publication at *PLOS ONE*. We have now prepared a revised manuscript for you for consideration as a Research Article.

Below, you will find our detailed Response to Referees. We have uploaded a manuscript with tracked changes for your review.

Editor and Reviewer 1 comment

All comments except one from Reviewer 1 and from the Editor were positive, noting that the "conceptual framing [is] very interesting" and that it was "a very nice study." The sole critique claimed that our study was unreplicated. This however is an incorrect interpretation of our methods.

The section from our methods describing the replication of our experiments was as follows.

"Experiment one used eight unique families, whereas experiment two used six families. After hatching, tadpoles were pooled by family and then haphazardly divided into treatment groups. In experiment one, we used 80 tadpoles from each family, set up in eight groups of 10 tadpoles each in 2 L of water. In experiment two, we used 76–88 tadpoles from each family, set up in four sets of approximately 20 tadpoles each in 4 L of water. Extra hatchlings were used to feed predators in order to generate cues for the experiments."

We apologize if the degree of replication was unclear. We thought that it would be clear given the number of animals being described in each experiment. For example, experiment one is described as having eight unique families, which 80 tadpoles used from each family, meaning 640 total tadpoles. If tadpoles were set up in groups of 10, that indicates 64 different units in the experiment, or eight full replicates of the eight treatments. The same logic applies to experiment two. However, in order to remove and prevent readers from similarly misinterpreting the design, we have modified the text of the methods as follows, with new text indicated in green:

"Experiment one used eight unique families, whereas experiment two used six families. After hatching, tadpoles were pooled by family and then haphazardly divided into treatment groups. In experiment one, eight replicates of the experiment were setup (i.e., each family was a complete

replicate of all treatments). We used 80 tadpoles from each family, set up in eight groups of 10 tadpoles each in 2 L of water. In experiment two, the experiment was once again replicated by family (i.e., all treatments were replicated six times). We used 76–88 tadpoles from each family, set up in four sets of approximately 20 tadpoles each in 4 L of water. Extra hatchlings were used to feed predators in order to generate cues for the experiments."

Thank you for your consideration.

Sincerely,
Justin C. Touchon
Associate Professor of Biology

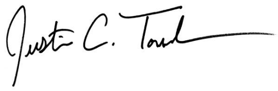

Supplement: Attachment — Submitted filename: Rosenthal etal v2 PLoS One Response to Reviewers.pdf [file pone.0285968.s001.pdf]
