# Supplementary material for: One of these things is not like the other: Mixed predator cues result in lopsided phenotypic responses in a Neotropical tadpole
Source: PLoS One. 2023 May 23;18(5):e0285968. doi: 10.1371/journal.pone.0285968 (PMC10204971; doi:10.1371/journal.pone.0285968)
Supplement: Attachment — Submitted filename: Rosenthal etal v3 PLoS One Response to Reviewers.docx [file pone.0285968.s002.docx]

April 7, 2023

Dr. Justin C. Touchon

Assistant Professor

Vassar College

Dr. Peter Eklöv

Professor at Department of Ecology and Genetics

Uppsala University

Dear Dr. Eklöv,

Thank you for the recent reviews of our manuscript “One of these things is not like the other: mixed predator cues result in lopsided phenotypic responses in a Neotropical tadpole” which was submitted for publication at *PLOS ONE*. We have revised the manuscript in accordance with the comments from yourself and the reviewers.

Below, you will find our detailed Response to Referees. We have uploaded a manuscript with tracked changes for your review.

**Editor comments**

We have updated our manuscript and figure files to follow the naming and style guidelines of PLOS One, including updating the author affiliations, and font sizes of level 1 and 2 headings. We have updated the Acknowledgements section to remove the funding statement and IACUC approval statement. The IACUC approval statement with approved protocol numbers has been placed at the beginning of the Materials and methods section. Our full funding disclosure is as follows:

Funding was provided by Vassar College and the Vassar College Diving Into Research program. Justin C. Touchon receives a salary from Vassar College. The funders had no role in study design, data collection and analysis, decision to publish, or preparation of the manuscript.

Note that there are no grant numbers to report with regards to the funding.

**Response to Reviewer 2**

Reviewer 2 made two comments, asking us to 1) soften our language in the Abstract about the responses of the tadpoles to mixed cues of our two predators and 2) to consider an addition reference in our Introduction. We have implemented both of these changes, which are shown below with new text in green.

Abstract, lines 42–46: “Previous studies have shown that fish are more lethal than dragonfly larvae; thus tadpoles responded most strongly to the more dangerous predator, even though the number of prey consumed by each predator was the same. This may be due to *D. ebraccatus* having evolved a stronger response to fish or because fish produce more kairomones than do dragonflies for a given amount of food.”

Introduction, lines 70–74: “Depending on the predator-prey relationship, the phenotype-inducing cue may be a combination of kairomones released from the predator alone, alarm pheromones produced by conspecific prey under attack, density based cues of conspecifics, and/or compounds and tissues derived from the mastication and digestion that occur with predation events [9-12].”

Thank you for your consideration.

Sincerely,

Justin C. Touchon
Associate Professor of Biology
